# Safety evaluation of visual load at entrance and exit of extra-long expressway tunnel based on optimized support vector regression

**DOI:** 10.1371/journal.pone.0272564

**Published:** 2022-08-04

**Authors:** Ting Shang, Hao Lu, Jiaxin Lu, Jing Fan

**Affiliations:** 1 School of Traffic & Transportation, Chongqing Jiaotong University, Chongqing, 400074, China; 2 School of Economics & Management, Chongqing Jiaotong University, Chongqing, 400074, China; Huazhong University of Science and Technology, CHINA

## Abstract

The traffic environment of an extra-long expressway tunnel is more complex than that of a long tunnel, which increases the driving risk. The visual load of drivers can be used to evaluate driving safety and comfort. To reveal drivers’ visual load characteristics at the entrance and exit of extra-long tunnels on mountainous expressways, this study conducted vehicle tests with 12 drivers at Gonghe extra-long tunnel on the Yu-Xiang expressway in the Wulong District. An eye tracker, non-contact multifunctional velocimetry, illuminometer, and other test equipment were used to record drivers’ pupil areas, velocity, and illuminance when entering and leaving the tunnel. The change characteristics of drivers’ pupil areas were studied. The maximum transient velocity value (MTPA) of the pupil area was selected as an index to evaluate the visual load degree. Based on velocity and illuminance coupling, a visual load model was constructed using the optimized support vector machine (GA-SVM). The influence of velocity and illuminance on the MTPA in the tunnel’s approach, entrance, exit, and departure section was analyzed. The results show that drivers’ psychological tension order at the entrance and exit is entrance section ≈ exit section > departure section > approach section. In the approach section, the visual load is mainly affected by environmental illumination. In the entrance and exit sections, the visual load is positively correlated with velocity and negatively correlated with illuminance, and velocity has a greater impact on visual load. In the tunnel departure section, the two variables synergistically influence the driving visual load. The research results provide theoretical support for the safety design and management of extra-long tunnel entrances and exits.

## 1. Introduction

With the development of highways in remote mountainous areas, tunnels, as important structures for mountain crossing, have increased in number and scale in recent years. By the end of 2021, China had 23,268 tunnels totaling 24.6989 million meters, including 1599 extra-long tunnels (i.e., a tunnel exceeding 3000 meters in length) totaling 7.1708 million meters, increases of 12.27% and 14.99%, respectively, over the previous year [1]. The traffic safety level of extra-long tunnels is worrying because of the high rate and intensity of traffic accidents, for which rescue is difficult. Driver’s dark adaptation time in the extra-long tunnel’s entrance section is longer than that of the long tunnel [2], and the average pupil area of the driver increases with the increase of the tunnel length to adapt to the low illumination environment in the tunnel [3]. Considering the great differences in illuminance, speed perception, reaction time, and visual range between extra-long tunnels and common road sections [4,5], how to improve their traffic safety is of great importance.

A tunnel has incomparable advantages in breaking through geographical barriers, shortening travel time, improving highway alignment, and protecting the environment, but the traffic safety situation is severe. Accident rates vary by section, and the entrance has the greatest risk, with an accident rate of 63.7%, which is 2–3 times that in the tunnel [6]. Studies have shown that sudden changes in the liner and the light environment combine with high speed at the entrance and exit to make it difficult for a driver to complete light and dark adaptation in sufficient time, which is also an important reason for the formation of black spots. In an extra-long tunnel, the complex driving environment, limited and relatively dark conditions, low visibility, uneven illumination, and the black- and white-hole effects make the cumulative superposition effect of the driver’s psychological pressure obvious, which causes an excessive visual load [7]. There is an inverted U-shaped relationship between a person’s work performance and physiological load [8]. A higher physiological load prolongs the driver’s reaction time, which can easily cause loss of control. Current highway engineering design and maintenance specifications lack safety design and management specific to extra-long tunnel entrances and exits.

To evaluate the safety of entrances and exits in extra-long tunnels from the driver’s perspective, 12 drivers were tested in the Gonghe extra-long tunnel of the Yuxiang Expressway. The maximum transient velocity of pupil area (MTPA) was selected as the characterization index. Based on velocity and illuminance coupling, the visual load model was constructed using the optimized support vector machine (GA-SVM). The influence of velocity and illuminance on MTPA in the approach, entrance, exit, and departure sections of the tunnel were analyzed. The findings of this work can provide a basis for safety design.

## 2. Literature review

### 2.1 Relationship between visual load and traffic safety

It has been found that the main tasks of drivers include perception of vehicle running environment, generation of decision control, and control of vehicles. The contribution rate of the driver’s vision to driving behavior is about 80% [9], and the visual load is closely related to the level of driving safety. Appropriate reduction of visual load is necessary for driving safety [10]. Most studies have used eye movement indexes such as pupil area, scanning speed, and blink to reflect a driver’s load change during driving. Reimer [11] found that the load level of driving tasks is closely related to pupil, blink rate, saccade amplitude, and fixation duration. Kim [12] found that panning is the only touch gesture that can be used without negative consequences when operating in-vehicle information systems with visual occlusion while driving. Ma [13] investigated the visual attention fixation and transition characteristics of drivers under different cognitive workloads. Bitkina [14] believed that Gaze point, duration, fixation, and pupil diameter significantly influence driving workload. Macdonald [15] believed that the visual load in tunnel driving is determined by the visual physiological and perception loads. Du [16,17] pioneered a driving visual load evaluation method based on pupil area’s changing rate and maximum transient velocity value of pupil area (MTPA). Researchers [18–21] used this index to study the influence of visual load changes on driving safety in long highway tunnels, subsea tunnels, extra-long tunnels, and high tunnel ratio sections. Yan [22] studied how light zones affect the dynamic visual characteristics and information perception of drivers as they pass through extra-long tunnels on highways. Li [23] analyzed the improvement of driving fatigue and driver’s attention by setting visual fatigue relief in extra-long tunnels.

Most studies have focused on the relationship between visual load and highway traffic safety. Literature on visual load and tunnel safety mainly studied highway and urban tunnels, with little attention to extra-long tunnels, whose traffic environment is quite different from that of other tunnels.

### 2.2 Driving safety in entrance and exit of expressway tunnel

Tunnel entrances and exits have a high incidence of traffic accidents, and the setting of traffic signs is crucial to traffic safety. To determine the location of speed limit signs in tunnel sections, Bai [24] considered the psychological influence of brightness changes of entrances and exits, selected the eye fixation time and rotation speed to characterize the psychological load and visual characteristics, and established a speed limit sign positioning model. Miller [25] evaluated stress as drivers traversed along an interstate route that included tunnel and non-tunnel segments, as well as a 75-m transition period before the tunnels. Shang [26,27] proposed a method to set exit advance guide signs in highway tunnels, and evaluated their influence on the trajectories and speeds of passenger cars. Zhu [28] evaluated the effectiveness of three types of speed reduction markings (SRMs) in tunnel entrance and exit zones through a driving simulation experiment, and found that SRMs are necessarily implemented in the highway tunnel entrance and exit zones. Rain and snow can lead to a bad line of sight, weaken anti-skiing ability, and aggravate risks at tunnel entrances and exits. In these areas, ice detection and prevention systems, as applications of intelligent transportation systems, can be used to reduce the number of traffic accidents. Alemdar [29] compared the advantages and disadvantages of conventional ice detection and prevention systems. Ma [30] proposed reasonable speed limits to reduce the number of traffic accidents on rainy days at tunnel entrances and exits, and showed that speed limits are needed when the rainfall intensity is greater than 1.5 mm/min. Wang [31] selected the maximum transient velocity value of pupil area (MTPA) as the visual load evaluation index, and found it to vary greatly in different regions of extra-long tunnel entrances and exits and in different time periods (morning, dusk, and night).

Scholars have studied the setting methods of traffic signs at tunnel entrances and exits and the influence of bad weather on these sections. A driver travels a long time in an extra-long tunnel in a narrow, closed area with limited vision, low visibility, and uneven brightness. The psychological load changes greatly, and the safety situation at the exit is serious. It is necessary to analyze the psychological state of the driver.

### 2.3. Support vector regression application in traffic field

The support vector machine (SVM) classification model is often used in traffic condition monitoring. Li found that the SVM classifier can be used to detect the traffic state of a road. Based on the data of urban road network adequacy, traffic flow, speed, and occupancy rate, Li [32] used an SVM model to identify five types of urban traffic states. Siddique [33] adopted vehicle trajectory data based on the Global Positioning System, and used SVM to identify stopping and forward behavior in a vehicle trajectory. Support vector regression (SVR) can be combined with other methods to predict traffic flow changes and accidents. Elamrani [34] combined an SVM model with a Bayesian learning machine to predict real-time traffic collision accidents. Basso combined SVR with logistic regression in a freeway accident detection model. Vishnu [35] utilized hybrid SVM (with an extended Kalman filter) in a traffic video surveillance and monitoring system with dynamic traffic signal control and an accident detection mechanism. Based on the real-time detection data of Yanan Expressway in Shanghai, Wang [36] constructed a short-term traffic speed prediction model by combining SVR with wavelet analysis to explore the relationship between traffic flow, traffic speed, and average occupancy.

In the field of traffic literature, SVM model was more used for accident classification, and SVR model is seldom used for prediction and influencing factor analysis. Unitary or binary regression analysis was widely used to analyse the influencing factors of tunnels. Due to the limitation of data and the coupling effect among influencing factors, it is difficult to pass the test for multiple regression model. Some recent literature has discussed the application of SVM to traffic safety from the perspective of driver behavior. To improve the signal operation efficiency of intersections, Moynur [37] combined linear SVM, polynomial SVM, and an artificial neural network to predict driver behavior under time and speed changes.

Hua [38] conducted a natural driving experiment with three representative events at a non-signalized intersection in a mixed traffic environment and proposed a method to identify cognitive distraction based on bidirectional long short-term memory (Bi-LSTM) with an attention mechanism, whose recognition accuracy rate was 3.84% higher than that of an SVM model. Chai [39] used a driving simulator to collect data and established an SVM rating model to detect the sleepiness of drivers. Gonzalez-Ortega [40] compared four methods of gaze estimation to learn the relation between gaze displacement and head movement. Two were simpler, and based on points that try to capture this relation, and two were based on MLP and SVM classifiers. Liao [41] compared the extracted optimal feature subsets and SVM performance of two typical driving scenarios. Analysis of a driver’s visual characteristics using SVM in mountain highway tunnels is still relatively rare.

In summary, scholars in the driver’s tunnel driving visual load characteristics and extra-long tunnel driving safety research, mainly for highway long tunnel and urban underpass tunnel research. However, there are significant differences between extra-long, long, and urban tunnels in terms of linear design, travel time, and visual area, and the cumulative effect of the long-term tunnel traffic visual load has not been considered. At the same time, there is a lack of quantitative research on the impact of brightness, speed, location, and other factors on driver visual load. Based on the experimental data of drivers in an extra-long tunnel, this study explores the coupling mechanism and influence law of extra-long tunnel entrance brightness, operating speed, and location on a driver’s visual load, so as to lay a theoretical foundation for the traffic safety design and management of extra-long tunnels.

## 3. Methods

### 3.1 Ethics statement

The study was approved by the College of Transportation, Chongqing Jiaotong University. All participants provided written informed consent. The early research content of the project strictly follows the ’ Declaration of Helsinki ’, ’the International Code of ethics for Biomedical Research Involving Human Beings’, and the relevant provisions of the College of Transportation, Chongqing Jiaotong University. In the implementation process of this project, the informed consent, processing scheme, and possible compensation methods will be strictly done to ensure that the personal information and medical information of the sample source are not disclosed publicly, and all efforts will be made to protect the privacy of the personal medical information, disease information, life information and genetic information of the sample source within the scope permitted by law.

### 3.2 Real vehicle test

**Fig 1:**
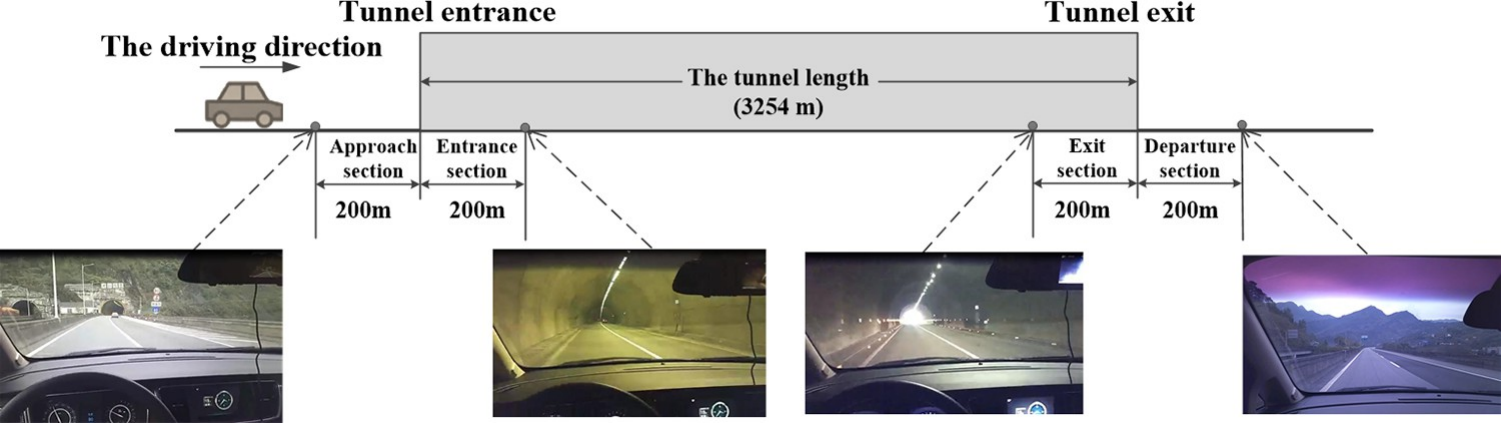
The tunnel section division.

To truly reflect the driver’s eye movement and driving characteristics in an extra-long tunnel, the Gonghe extra-long tunnel of Yuxiang Expressway was taken as a real vehicle test tunnel, with a length of 3254 m and a speed limit of 80 km/h. This double-hole one-way four-lane tunnel has an average longitudinal slope of less than 2%. There are no other tunnels, bridges, interchanges, or service areas before or after the tunnel. The two-way lanes outside the tunnel are separated by a green belt, and the surrounding mountains mainly present green vegetation. There is little difference in the operating environment of the whole test section. To reduce the interference of traffic volume, the test time ran from Tuesday to Thursday, 10:00–16:00. The change of light environment outside the tunnel has a great influence on the driver, so the test was conducted in fine weather. Traffic volume was in a free flow state during the test. Considering the literature[16,20], the test tunnel was divided into four sections (Fig 1): approach section L_1_ (200 m before the entrance), entrance section L_2_ (200 m after the entrance), exit section L_3_ (200 m before the exit), and departure section L_4_ (200 m outside the exit).

### 3.3 Apparatus and tools

**Fig 2:**
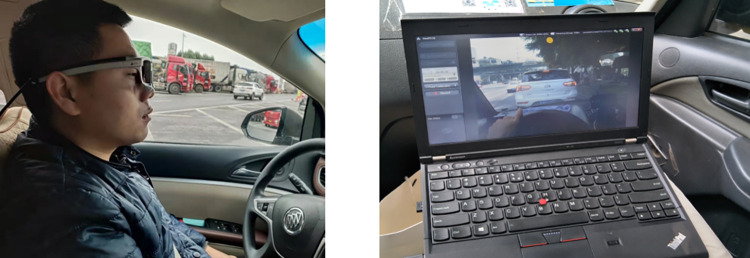
Eye tracker.

**Fig 3:**
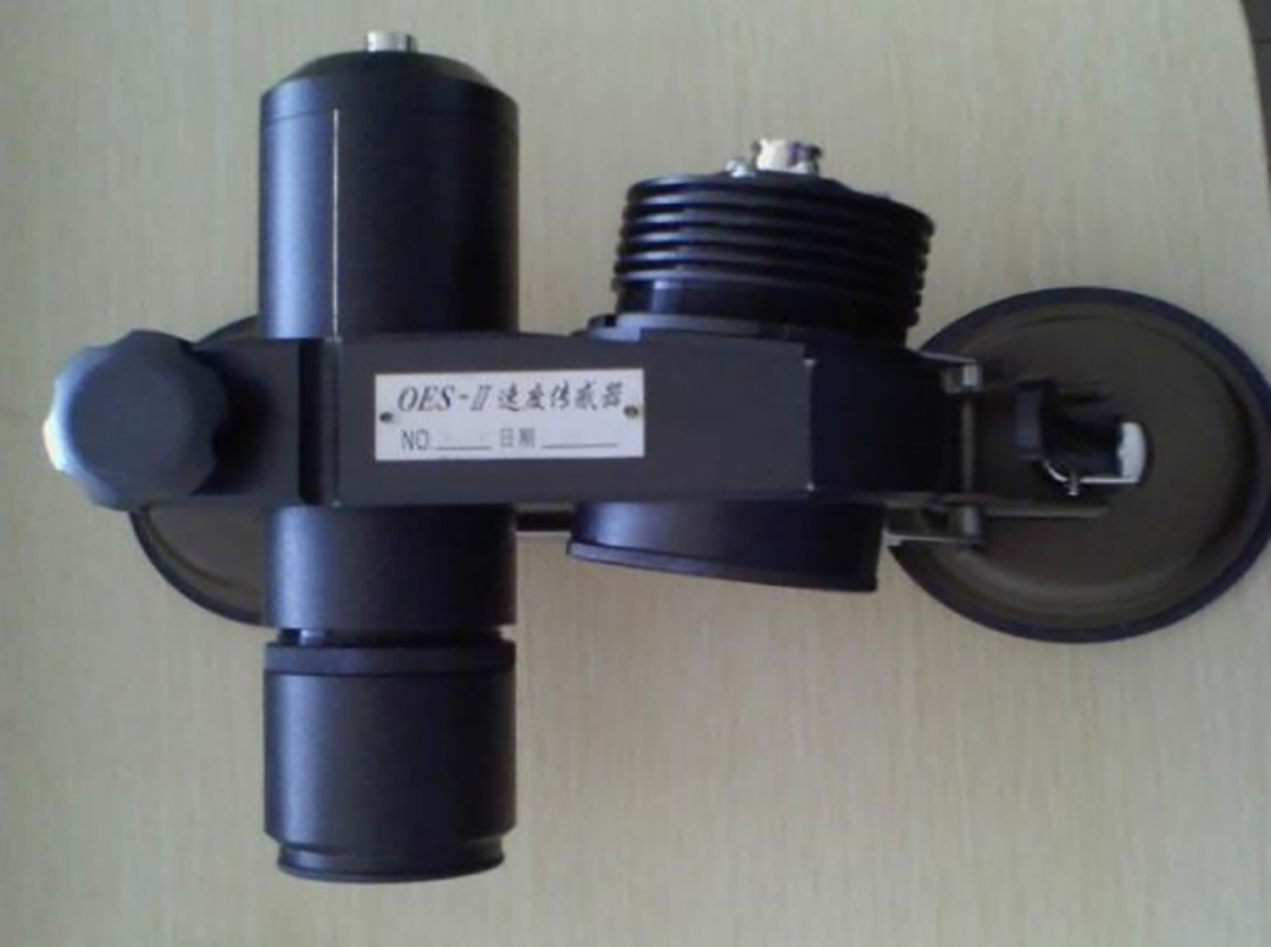
Non-contact velocimetry with multi-function.

**Fig 4:**
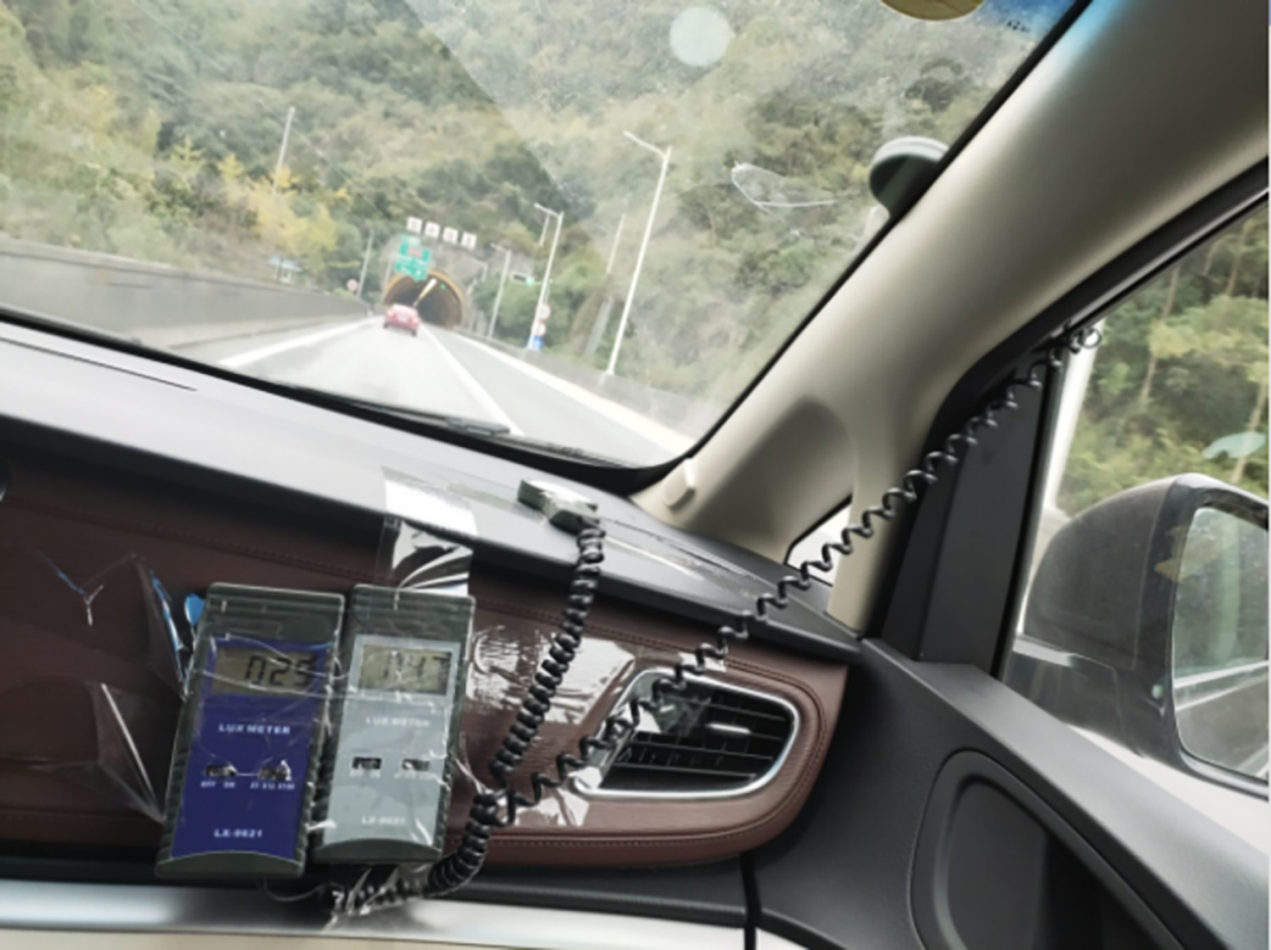
Illuminometer.

Small cars are the main vehicles in the test section. This test required multiple recorders, so seven commercial vehicles in Buick GL8 were selected as the test vehicles. The SMI2.1 glasses eye tracker (SMI, Germany) was used to acquire eye movement data, with 60 Hz sampling frequency and 0.1° tracking accuracy as shown in Fig 2 The horizontal and vertical tracking ranges of the camera were 60 deg and 46 deg, respectively. Vehicle speed acquisition was realized by a non-contact multi-function speedometer with measurement range 0–300 km/h and 0.1 km/h accuracy as shown in Fig 3. The LX-9621 illuminometer (Tasi, China) acquired illumination data, with measurement range 0–50000 Lux and precision ±3% rdg ±0.5% f.s as shown in Fig 4.

### 3.4 Participants

To better represent the regular characteristics of the driver group, this paper strictly follows “minimum sample size” principle in statistics [42]. The required minimum sample size was 10 in our test. Twelve participants were recruited, including nine males (six aged 22–40, three aged 41–60) and three females (aged 22–45). The gender ratio was consistent with the statistical characteristics of Chinese drivers [43]. Considering the risk of a real vehicle test, subjects were required to have more than 3 years of highway driving experience, corrected visual acuity of more than 5.0, and a Chinese driving license. Before the test, drivers were informed to drive according to their driving habits, comply with traffic rules, and pay attention to safety. The driver rotation test was adopted to ensure that each driver underwent at least two effective tests and that the speed limit was not exceeded. The standard physical conditions should be maintained before the test. The individual pictured in Fig 2 has provided written informed consent (as outlined in PLOS consent form) to publish their image alongside the manuscript.

### 3.5 Measures and statistical analyses

The entrance section of the long tunnel serves as the starting point for the research and analysis of the changes of pupil, driving speed, and illumination in the process of entering and leaving the tunnel. The collected pupil data were extracted using BeGaze3.5 (SMI, Germany) data analysis software, and abnormal noise data with high dispersion were eliminated according to the Riyadh criterion. SPSS, ORIGIN, and other software were further used to analyze the eye movement, illumination, and velocity data. To study the influence mechanism of driving speed and illumination change on drivers’ visual load in the entrance and exit section of the tunnel, a visual load model based on the coupling effect of driving speed and environmental illumination was constructed using optimized support vector machine (GA-SVM), and the relationships between visual load and various influencing factors were quantitatively analyzed.

#### 3.5.1 Support vector regression (SVR)

SVR is a nonlinear regression method based on a sum function. It attempts to find the best regression hyperplane with the least structural risk in the so-called high-dimensional feature space.

Given training data $\{\left(x_{i},y_{j}\right)\},i,j=1,2,\cdots ,n$, where *x*_*i*_ is the input vector and *y*_*i*_ is the corresponding output value, *x*_*i*_ is mapped to *f* by selecting the appropriate kernel function *f*, and linear regression is conducted on

$$f(x)=\omega^{T}\varphi (x_{i})+b$$ (1)

where *ω* is the weight vector of the hyperplane and b is the characteristic constant. SVM solves the problems

$$\underset{\omega ,b,\xi ,\xi_{i}^{'}}{\min}J=\frac{1}{2}\omega^{T}\omega +C\sum_{i=1}^{n}\left(\xi_{i}+\xi_{i}^{'}\right)$$ (2)

with constraints

$$s.t\{\begin{array}{l} y_{i}-\omega^{T}\varphi (x_{i})-b\leq \varepsilon +\xi_{i}\\ \omega^{T}\varphi (x_{i})+b-y_{i}\leq \varepsilon +\xi_{i}^{'},i=1,2,\cdots ,n\\ \xi_{i},\xi_{i}^{'}\geq 0 \end{array}$$ (3)

where $\xi_{i},\xi_{i}^{'}$ are relaxation variables representing the upper and lower limits, respectively, of training error under the error constraint *ε*. The cost function is 0 when the predicted value is within *ε*, and otherwise is the magnitude of the predicted value minus *ε*. The penalty coefficient *C*>0 reflects the penalty degree of the sample when the error *ε* is exceeded.

To solve the above problems, based on the optimal conditions of KKT, the Lagrange function is

$$\begin{array}{l} L=\frac{1}{2}\omega^{T}\omega +C\sum_{i=1}^{n}\left(\xi +\xi^{'}\right)-a_{i}\sum_{i=1}^{n}\left(\xi_{i}+\varepsilon -y_{i}+\omega \cdot x_{i}+b\right)-\\ a_{i}^{'}\sum_{i=1}^{n}\left(\xi_{i}^{'}+\varepsilon +y_{i}-\omega \cdot x_{i}-b\right)-\sum_{i=1}^{n}\left(\eta \xi_{i}-\eta_{i}^{'}\xi_{i}^{'}\right) \end{array}$$ (4)

where $a_{i},a_{i}^{'},\eta_{i},\eta_{i}^{'}>0$ is a Lagrange multiplier. According to the KKT optimization conditions, the following equations should be satisfied:

$$s.t\{\begin{array}{l} \sum_{i=1}^{n}(a_{i}-a_{i}^{'})=0\\ 0\leq a_{i},a_{i}^{'}\leq C\\ \omega =\sum_{i=1}^{n}\left(a_{i}-a_{i}^{'})\varphi (x_{i}\right) \end{array}\;i=1,2,\cdots n$$ (5)

Therefore, the nonlinear regression problem can be solved by solving the dual problem of (2) as

$$\begin{array}{l} \underset{a,a^{'}}{\max}W(a_{i},a_{i}^{'})=-\frac{1}{2}\sum_{i,j=1}^{n}\left(a_{i}-a_{i}^{'}\right)(a_{j}-a_{j}^{'})k(x_{i},x_{j})-\\ \varepsilon \sum_{i=1}^{n}\left(a_{i}-a_{i}^{'}\right)+\sum_{i=1}^{n}y_{i}(a_{i}-a_{i}^{'}) \end{array}$$ (6)

where the key to controlling the fitting function is $0\leq |\left(a_{i}-a_{i}^{'}\right)|\leq C$, where *x*_*i*_ is a support vector. The kernel function $k(x_{i},x_{j})=\varphi {\left(x_{i}\right)}^{T}\cdot \varphi (x_{j})$ is the inner product of the high-dimensional feature space. The kernel function must meet the Mercer condition. We solve for *a*_*i*_ and $a_{i}^{'}$, and substitute them into (5) to obtain the regression function from (1),

$$f(x)=\sum_{i=1}^{N}\left(a_{i}-a_{i}^{'}\right)k(x,x_{i})+b$$ (7)

where N is the number of support vectors, and *K*(*x*,*x*_*i*_) is the kernel function, which in this study is the Gaussian kernel function,

$$k(x,x_{i})=\exp(\frac{{\left\|x-x_{i}\right\|}^{2}}{\sigma^{2}})$$ (8)

#### 3.5.2 The genetic algorithm (GA)

The genetic algorithm (GA) is commonly used in evolutionary optimization, which depends on the selection of parameters, encoding the solution of the target problem into chromosomes, and constantly selecting, crossing, and mutating to produce new individuals. GA can be used to improve the SVR model, constantly search its optimal parameters (kernel function parameter g and penalty parameter C), and establish the error model with the best effect. The optimization algorithm is as follows:

1) Normalize the obtained index data,

$$x_{ij}^{'}=\frac{x_{ij}}{\max\{x_{ij}\}}$$ (9)

2) Construct the initial population and encode the chromosome of the objective function (kernel function parameter *g* and penalty parameter C);
3) Calculate the fitness value, which is used to evaluate the advantages and disadvantages of a population, which is convenient for individual selection. The absolute value of the deviation between the error prediction value *y*_*i*_ and the error measured true value *Y*_*i*_ is used in the fitness function,

$$H=g(\sum_{i=1}^{n}abs(y_{i}-Y_{i}))$$ (10)

4) Use the roulette method to randomly select individuals *i* from the original population for population reorganization. The individual selection probability is

$$P_{i}=\frac{H_{i}}{\sum_{i=1}^{n}H_{i}}$$ (11)

5) Select two individuals from the original population for cross-recombination. The new generation of individuals contains the excellent characteristics of the previous generation. The crossover pattern of chromosomes *a*_*k*_ and *a*_*h*_ at position *i* is

$$\begin{array}{l} a_{ki}=a_{ki}(1-b)+a_{hi}b\\ a_{hi}=a_{hi}(1-b)+a_{ki}b \end{array}$$ (12)

6) Perform mutation operation, which can obtain genes that are not present in the original population, adding new individuals to the population. Mutation operation keeps the diversity of gene types in the population, expands the search space, and prevents the system from falling into the local optimal cycle. In the real world, the mutation probability of biological genes is relatively low, so the mutation probability Pm is set in the range of 0.0001–0.1. Here, the mutation probability Pm was set to 0.01.

## 4. Results

### 4.1 Characteristics of pupil area change rate

**Fig 5:**
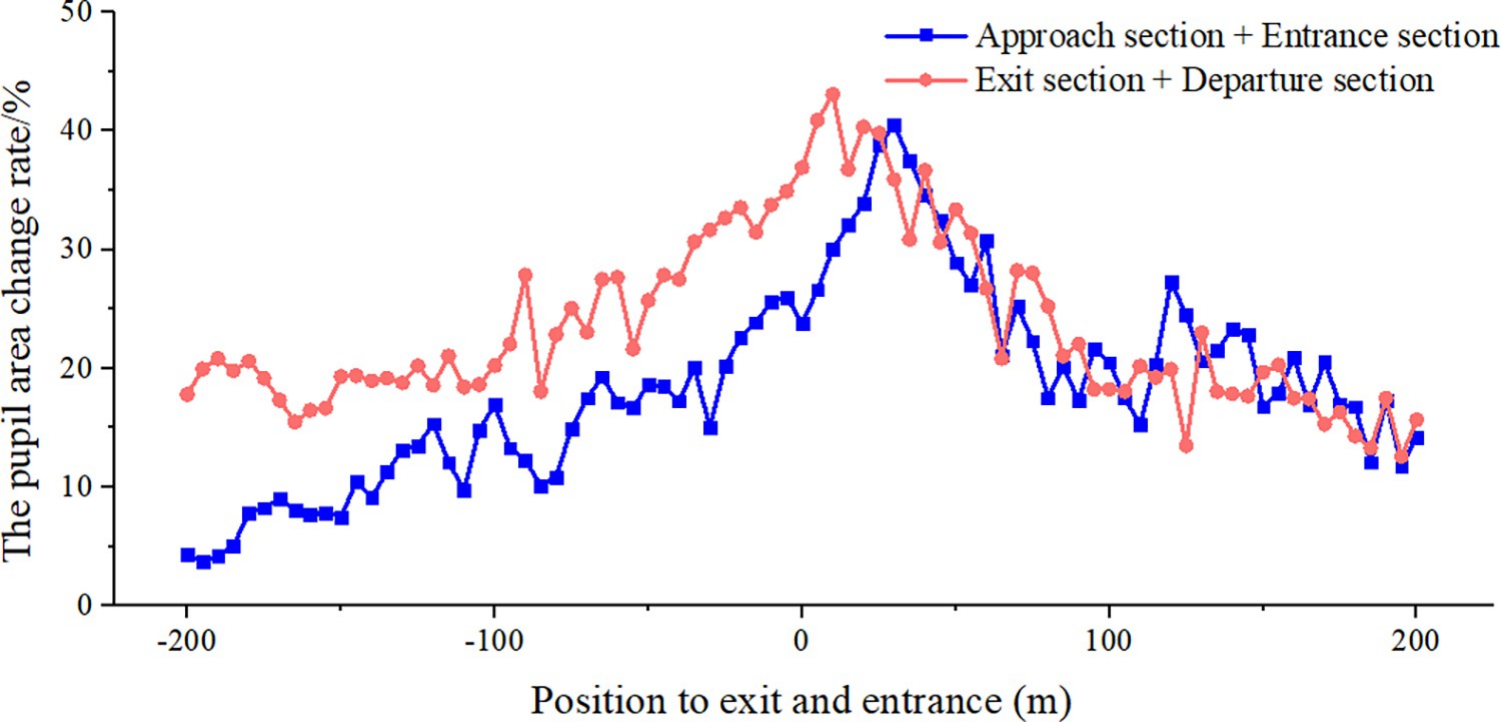
The pupil area change rate at entrance and exit of extra-long expressway tunnel.

Pupil size is a sensitive indicator of human physiological and psychological loads, so its change can objectively reflect the physiological and psychological states of drivers. The pupil area change rate at the entrance and exit of the extra-long tunnel was obtained as the average effective data of 12 drivers collected from the real vehicle test, as shown in Fig 5.

The pupil area shows an obvious change trend in Fig 5. Before the tunnel entrance, 200 m–100 m, the pupil area change rate is concentrated between 10% and 20%, showing a slow upward trend. It changes sharply 100 m before the tunnel entrance, where it reaches 26%, and 30 m after the entrance, it climbs until it reaches a peak at 40.48%. The pupil area change rate then starts to decrease to less than 20% at 100 m after the tunnel entrance. At 200 m before the exit, it maintains a relatively stable trend, fluctuates slightly within 20%, and is higher than at 200 m before the tunnel entrance. At 100 m before the exit, the pupil area change rate starts to increase, reaching 36.90% at the tunnel exit. After driving out of the tunnel, it still shows a slight increase, reaching 43.08%, and then beginning to fall back. After 100 m from the exit, it basically falls below 20% and remains at about 15%.

### 4.2 Characteristics of drivers’ visual load

When driving into and out of the tunnel, the sudden change of the light environment leads to a sharp change in the driver’s pupil area, and the change rate increases rapidly. Research has shown that the maximum transient velocity of the drivers’ pupil area can be used to evaluate the visual load at the entrance and exit of the tunnel.

#### 4.2.1 Visual load representation

The driver’s pupil area changes rapidly with the sudden change of the light environment at the entrance and exit of the tunnel, which is a transient motion with a rapid attenuation of amplitude and a short duration. Therefore, the weighted root-mean-square acceleration can be used to evaluate the instantaneous vibration of the human body according to *Mechanical Vibration and Shock* (ISO 2631-1-1997). Therefore, the continuous weighted root-mean-square velocity of the pupil area,

$$Ve_{k}(t_{0})={\left[\frac{1}{f}\int_{t_{0}-f}^{t_{0}}V_{w}^{2}(t)dt\right]}^{\frac{1}{2}}$$ (13)

can be used to evaluate the visual load, where *Ve*_*k*_(*t*) is the maximum transient velocity of the pupil area, *f* is the continuous average integral time, *t* is the time (integration variable), *t*_*0*_is the inspection time (transient), and *V*_*w*_ is the rate of pupil area change.

The maximum transient velocity value of the pupil area [7] is

$$Ve_{MTPA}=\max\{Ve_{k}(t_{0})\}$$ (14)

When measuring the tunnel entrance and exit, *f* takes the recommended value 1 s, and the unit for evaluating the tunnel entrance and exit visual load is *mm*^*2*^*/s*.

#### 4.2.2 Evaluation results

The duration of visual shock is generally between 0.05 s and 1.00 s at the entrance and exit of the tunnel. To quantify the visual psychological and physiological load of drivers, the converted duration of visual shock is used as an indicator to evaluate the visual load of drivers [7,13], to obtain the visual comfort in the entrance and exit sections, as shown in Table 1.

**Table 1:** Relationship between *MTPA* and visual comfort.

*MTPA*(mm^2^·s^-1^)	Entrance section	<20	20–30	30–70	70–105	≥105
	Exit section	<30	30–40	40–85	85–105	≥105
Converted duration of visual shock *t*_*c*_(s)		≤0.1	0.1–0.2	0.2–1.0	1.0–1.5	>1.5
Subjective feelings		Comfort	Slightly uncomfortable	Uncomfortable	Very uncomfortable	Extremely uncomfortable

According to Table 1, the MTPA of drivers is less than 20 mm^2^/s from 200 m in front of the tunnel entrance to 100 m behind the entrance, without discomfort. Within 100 m–200 m behind the entrance, MTPA exceeds 20 mm^2^/s, which makes vision slightly uncomfortable. Within 100 m after the exit, the average value of MTPA is 31.16 mm^2^/s, and the maximum value is up to 43.23 mm^2^/s, which is uncomfortable. Within 100 m–200 m after the exit, the average MTPA is reduced to 20.60 mm^2^/s, and the maximum value is 33.27 mm^2^/s, which is slightly uncomfortable.

### 4.3 Influence law of driving speed and environmental illumination on visual load

The driver’s visual load is closely related to driving speed and environmental illumination. Therefore, we studied this at the entrance and exit based on the change of driving speed and environmental illumination. The visibility test of real vehicle experimental data showed a significant influence of driving speed and environmental illumination on visual load. Origin software was used to analyze the relationship between velocity, illuminance, and visual load.

#### 4.3.1 Characteristic analysis of visual load change

**Fig 6:**
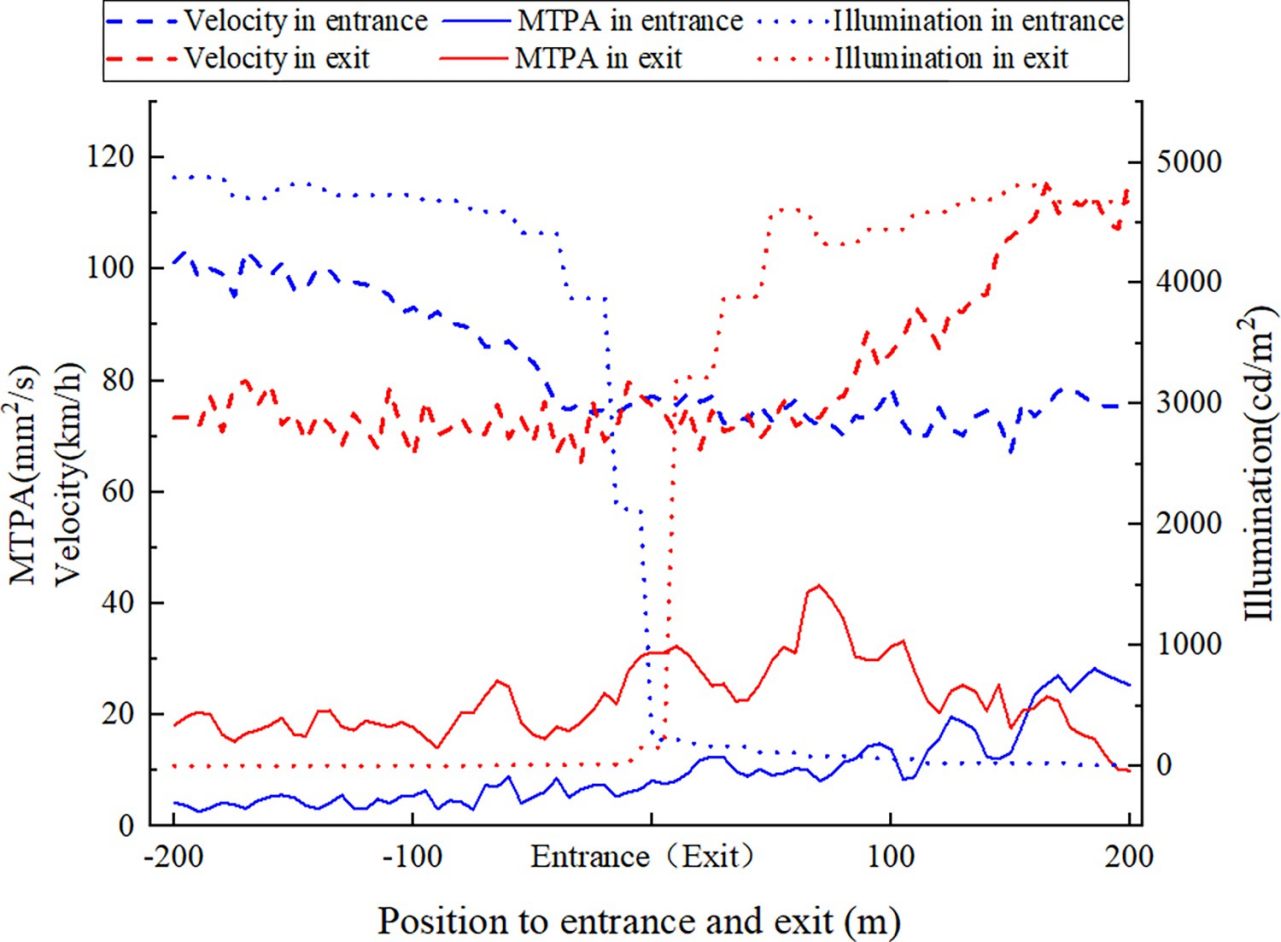
Velocity, illumination and *MTPA* of driver when passing through the extra-long tunnel.

Fig 6 shows the variation law of driving speed, environmental illumination, and visual load when driving through the entrance and exit of the tunnel.

In Fig 6, 200 m before the entrance of the tunnel, the driving speed decreases from 103 km/h to 75 km/h, the environmental illumination decreases sharply to 216 cd/m^2^ near the entrance, and the visual load shows an upward trend. In the range of 200 m after the entrance, the fluctuation of the driving speed is small, the environmental illumination continues to decline to 8.4 cd/m^2^, and the driver’s visual load continues to rise. At 200 m before the exit, the speed is maintained at about 73 km/h, the environmental illumination gradually increases, and the visual load increases to 30.54 mm^2^/s, which is higher than at the entrance. When driving 100 m out of the tunnel, the driving speed is maintained at 75 km/h, the environmental illumination rises to 4580 cd/m^2^ near the exit, and the driver’s visual load increases slightly first, and then decreases rapidly, with a peak of 43.23 mm^2^/s. When driving 100 m–200 m out of the tunnel, the speed rises to 116 km/h, the environmental illumination changes little, and the driver’s visual load decreases steadily.

#### 4.3.2 Influence of driving speed and environmental illumination on visual load

**Fig 7:**
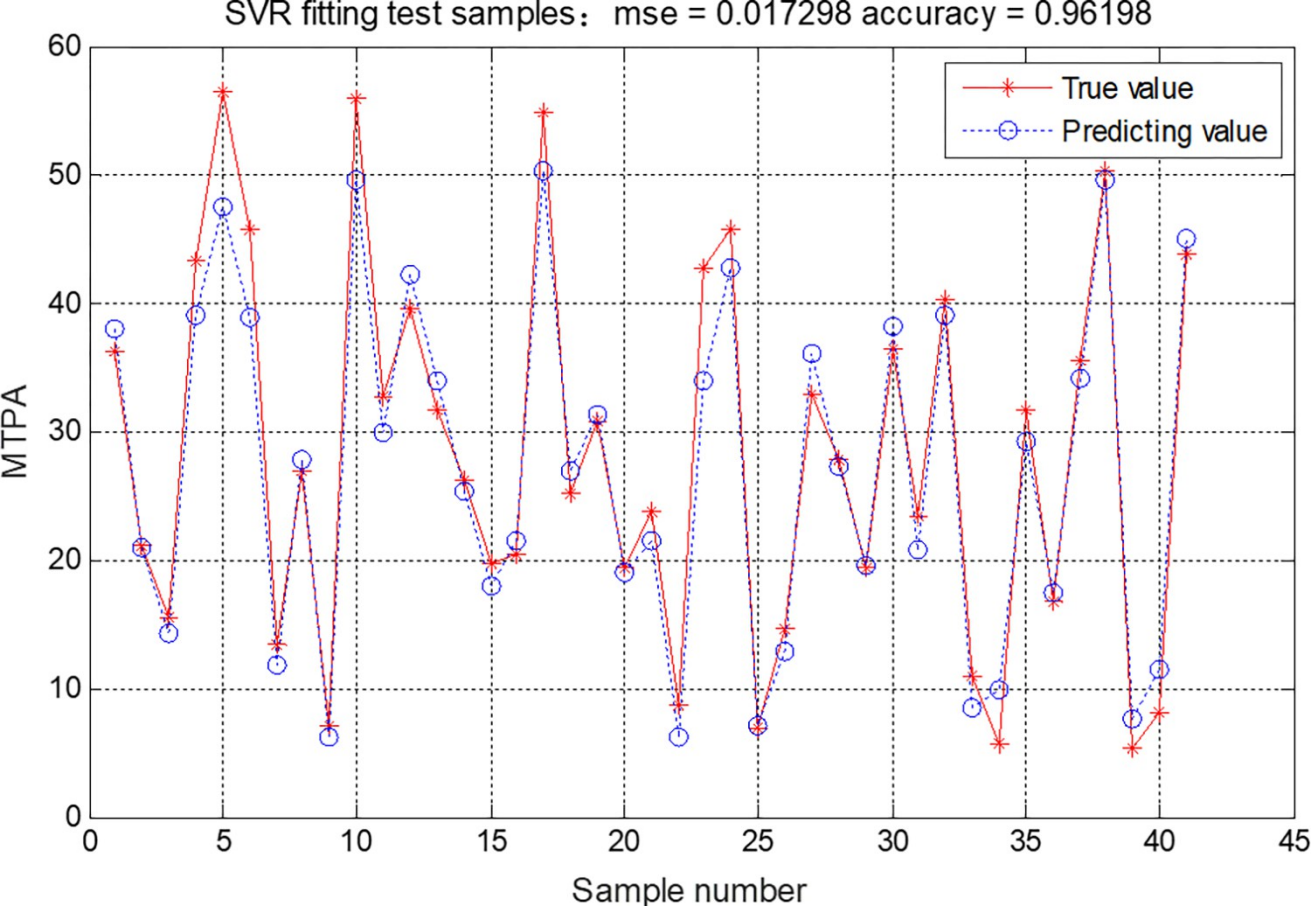
Predicting results of SVR.

*(1) GA-SVR model construction*. To further quantitatively study the influence of velocity and illuminance on MTPA, a GA-SVR model to calculate MTPA was established by Formulas (1)–(8). Firstly, velocity, illuminance, and position were taken as input variables *x*_*i*_, and MTPA was taken as output variables *y*_*i*_. Secondly, the parameters were set in the process of obtaining an SVR model by a genetic algorithm, where the evolution time was set to 500, the population size was set to 60, the mutation probability parameter was set to 0.05 and the crossover probability parameter was set to 0.4. In addition, the optimization range of penalty parameter C was set to [0.1,1000],and optimization range of kernel function parameter g was set to [0.01,1000]. 122 samples were randomly selected from162 valid samples as the training set, and the remaining 40 samples constituted the test set. To minimize the mean square error, the fitness function was established to search for the optimal penalty parameter and variance parameters in the SVR model. After inputting the optimal parameters to the SVR model and calling the LIBSVM toolbox in MATLAB, the optimal model was obtained after 500 iterations, and the MSE converges after 15 evolutions, and the minimum value is 0.012. At that time, *C* and *σ*^2^ are 17.5126 and 13.712, respectively. The results show that the MSE between the predicted and actual values was 0.017298, and the determination coefficient was 0.96198. Hence, the velocity and its influence could explain 96.20% of the variation of MTPA, and the GA-SVR model can therefore be considered to apply well to the calculation of MTPA, with results as shown in Fig 7.

**Fig 8:**
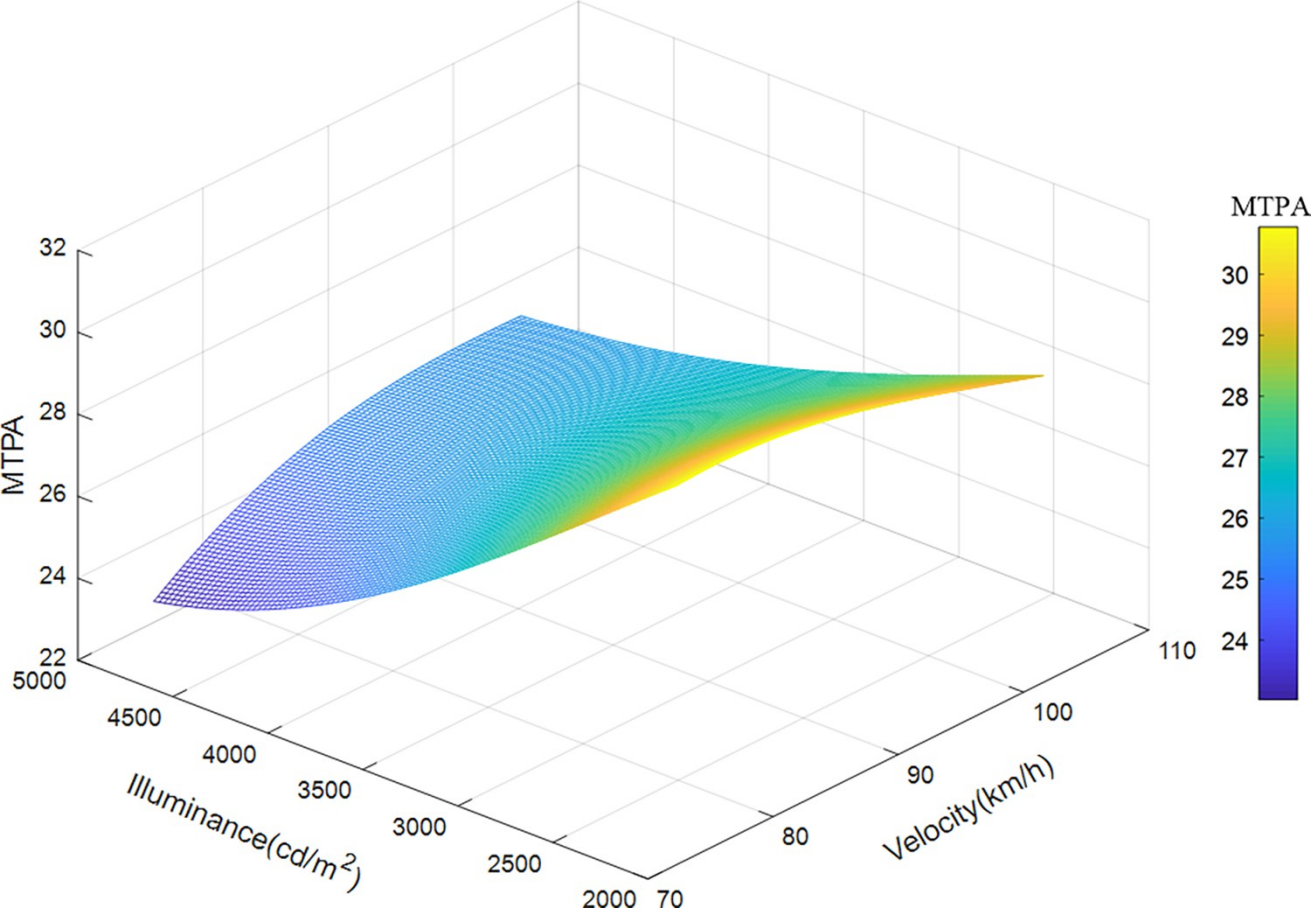
Variation characteristics of velocity, illuminance, and visual load in approach section.

*(2) Variation characteristics of velocity and illuminance*. Taken the velocity, illuminance, and position of the sample data of road section 1 (200 meters before the entrance) as the input variables, and MTPA as the output variable, the illuminance, and speed of this road section are divided into 100 parts, and the median of the position is taken, using the already trained The SVR model is run to obtain a 3D map of MTPA, as shown in Fig 8. Fig 8 shows the impact of driving speed and environmental brightness on the driver’s visual load when passing through the approach section of the tunnel. The visual load before the tunnel entrance decreases with the increase of illuminance, and the change of velocity has little effect on it. The predicted value of visual load varies in the range of 19.98–23.50 mm^2^/s, reaching a maximum when the speed is 75 km/h and the illuminance is 3700 cd/m^2^, and a minimum when the speed is 104 km/h and the environmental brightness is 3800 cd/m^2^.

**Fig 9:**
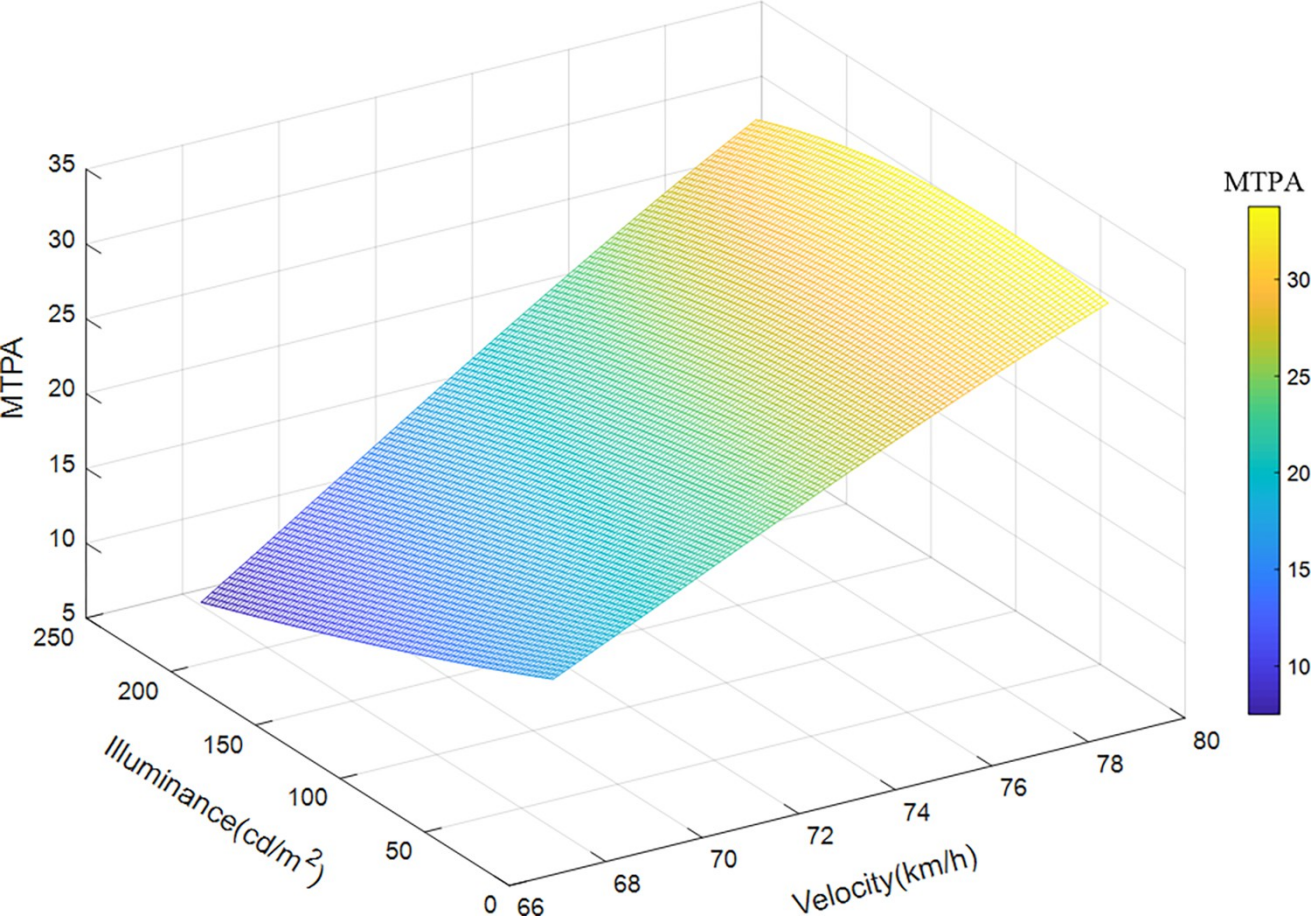
Variation characteristics of velocity, illuminance, and visual load in entrance section.

Taken the velocity, illuminance and position of the sample data of road section 2 (200 meters after import) as the input variables, and MTPA as the output variable, the illuminance and speed of this road section are divided into 100 parts, and the median of the position is taken, using the already trained The SVR model is run to obtain a 3D map of MTPA, as shown in Fig 9. Fig 9 shows the influence of velocity and illuminance on the driver’s visual load when passing through the entrance section. The overall visual load is positively correlated with driving speed and negatively correlated with environmental brightness. Changes in the former have a more pronounced effect on visual load. When the speed is 67 km/h and the environmental brightness is 220 cd/m^2^, the visual load reaches the maximum value of 33 mm^2^/s. When the speed is 80 km/h and the environmental brightness is 100 cd/m^2^, the visual load reaches the minimum.

**Fig 10:**
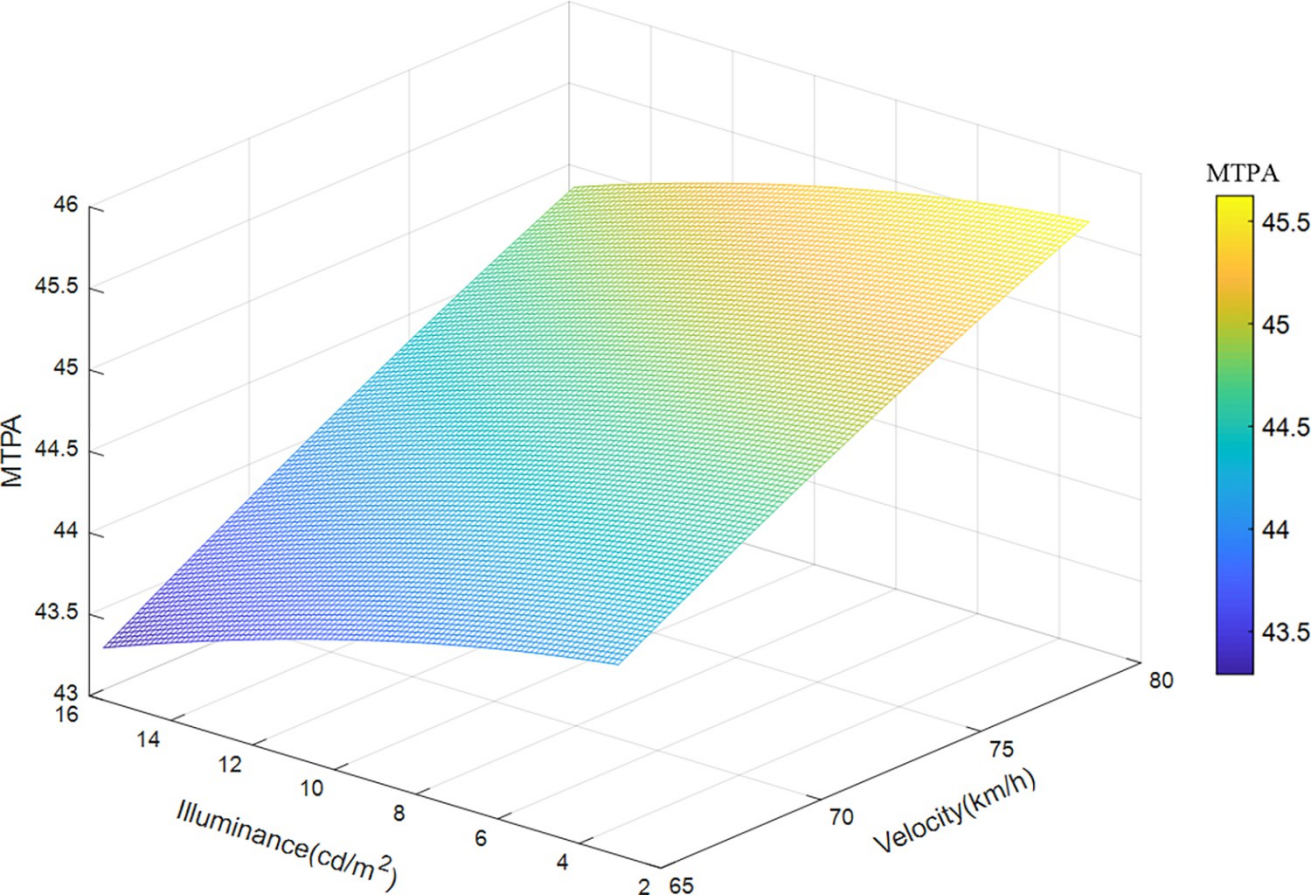
Variation characteristics of velocity, illuminance, and visual load in exit section.

Taken the velocity, illuminance, and position of the sample data of road section 3 (200 meters before the exit) as the input variables, and MTPA as the output variable, the illuminance and speed of the road section are divided into 100 parts, and the median value of the position is taken, using the trained The SVR model was run to obtain a 3D map of MTPA, as shown in Fig 10. Fig 10 shows the influence of driving speed and environmental brightness on the driver’s visual load when passing through the exit section. The visual load before the tunnel exit increases with driving speed and decreases with the decrease of environmental brightness. The influence of driving speed on visual load is dominant. The predicted value of the visual load varies from 43.3 mm^2^/s to 45.6 mm^2^/s, which is higher than that of the tunnel approach and entrance. When the speed is 65 km/h and the environmental brightness is 16 cd/m^2^, the driver’s visual load reaches the maximum. When the speed is 80 km/h and the environmental brightness is 3 cd/m^2^, the visual load reaches the minimum.

**Fig 11:**
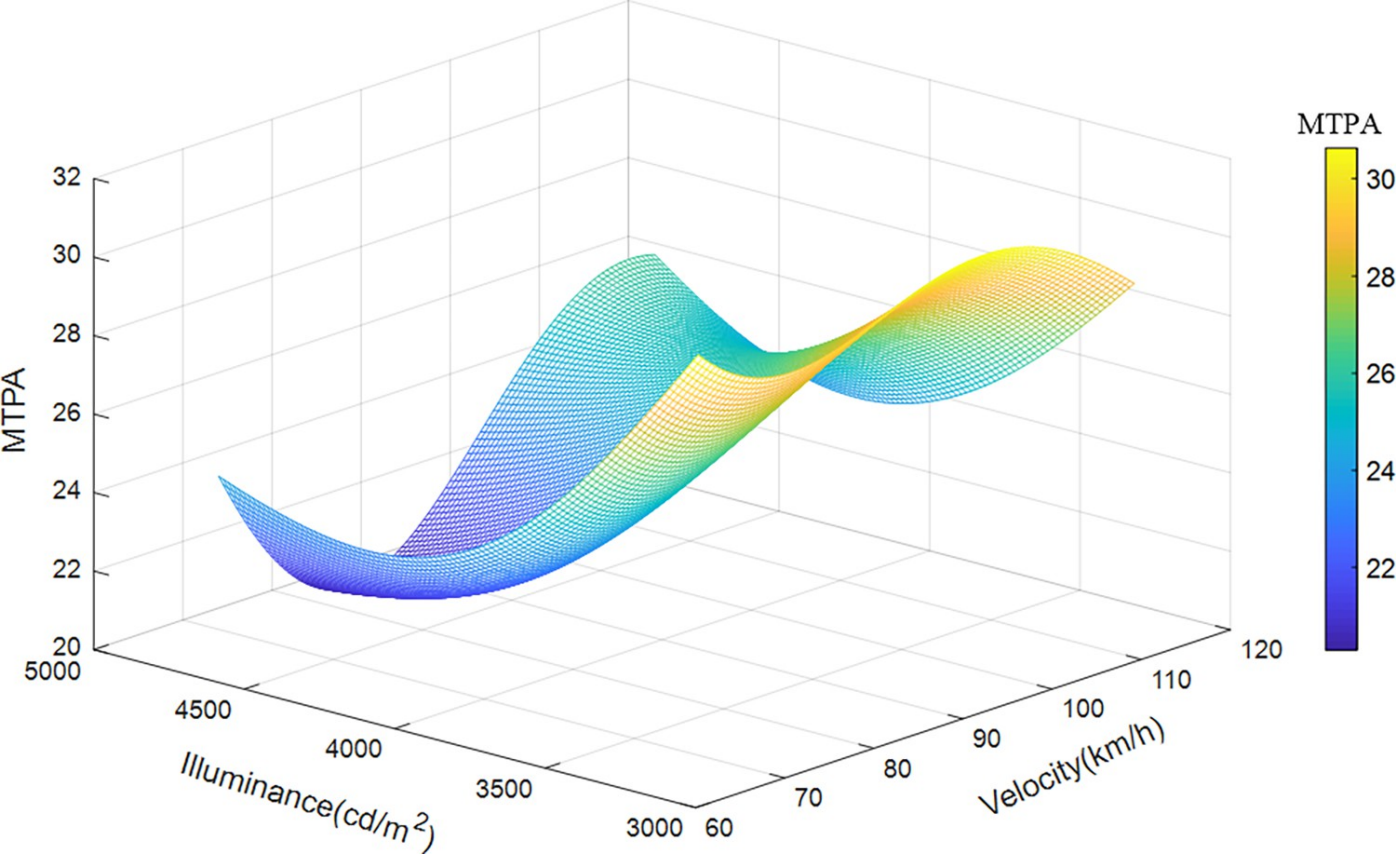
Variation characteristics of velocity, illuminance, and visual load in departure section.

Taken the velocity, illuminance, and position of the sample data of road section 4 (200 meters after the exit) as the input variables, and MTPA as the output variable, the illuminance and speed of the road section are divided into 100 parts, and the median value of the position is taken, using the already trained The SVR model was run to obtain a 3D map of MTPA, as shown in Fig 11. Fig 11 shows the influence of velocity and illuminance on the driver’s visual load when passing though the drive-away section. The change of visual load in this section is complex, and the influence of the two variables on the visual load is synergistic. The visual load decreases first and then increases with increasing velocity and illuminance. The predicted visual load varies from 20 to 34.5 mm^2^/s. The visual load reaches the maximum at a speed of 120 km/h and brightness of 3200 cd/m^2^. The driver feels most relaxed at a driving speed of 75 km/h and brightness of 4200 cd/m^2^, which differs from a long tunnel, whose visual load generally rises at the beginning due to the white-hole effect, decreases rapidly, and increases with speed.

## 5. Discussion

We evaluated the safety of drivers’ visual loads at the entrance and exit of an expressway tunnel. The change rate of the pupil area was concentrated between 10% and 20% at 200 m–100 m before entering the tunnel, and the driver was relatively comfortable [6]. At 100 m before the entrance, ow to the linear change and black-hole effect, the tension began to increase until 50 m after the entrance, and the driver gradually adapted to the tunnel environment. Owing to the confined environment, weak light, noise reverberation, and tail gas accumulation in the tunnel, after driving for a long time, the driver’s discomfort intensified, and at the 200 m–100 m section before the tunnel exit, the pupil area change rate was close to 20%, higher than at the tunnel entrance. In the exit section, owing to the driver’s long-term stress accumulation and the white-hole effect, the pupil area change rate exceeded 43.08%, slightly higher than the maximum at the tunnel entrance section. This is slightly different from the trend of tunnels of other lengths. Visual discomfort before the exit is generally more than after the exit [44], and the black-hole effect is stronger than the white-hole effect. However, owing to the longer driving time in an extra-long tunnel, the driver is more affected by environmental factors, and the cumulative effect of discomfort is obvious. Simultaneously, coupled with the synergy of the white-hole effect, although tension is effectively released at the exit, the change rate of the pupil area is still high. The order of mental stress in the exit section is tunnel entrance section ≈ tunnel exit section > tunnel departure section > tunnel approach section.

In the study of the driver’s visual load, it was found that MTPA increased first, and decreased as the driver passed through the tunnel. The visual feel of the driver in the approach, entrance, exit, and departure sections were comfortable, slightly uncomfortable, uncomfortable, and slightly uncomfortable, respectively. In particular, the driver’s visual load before the exit of the extra-long tunnel was higher than after the entrance. It is worth mentioning that this law is different from that of a long tunnel. The driver’s visual load in an extra-long tunnel is higher than that of a long tunnel, which indicates that long-term driving in the tunnel causes the accumulation of discomfort, resulting in an increased visual load, which is similar to the change rate of the pupil area.

In this paper, vehicle test was conducted in mountain tunnels, and driver visual characteristic data was obtained through wearable devices. Based on the Rare and expensive experimental data, GA-SVR method is combined to quantitatively study the influence of velocity, illuminance, and position on MTPA. The results showed that the prediction accuracy is 96.20%, which is the innovation of this paper. Referring to the contribution in methodology, this paper is based on the data from the sections of approach, entrance, exit, and departure in the tunnel, adopted GA method to obtain the optimization parameter value of SVR method. This deep learning method based on experimental data can objectively reflect the data rules and is more stable and accurate than ordinary GA-SVR method where the initial values were set in advance [45,46].

When a driver approaches an extra-long tunnel, the visual load decreases as illuminance increases, and the change of velocity has little effect on it. According to the relevant design specifications [47], the speed limit sign is set at 200 m before the tunnel. The vehicle keeps a speed below 80 km/h before the entrance and maintains it at the tunnel entrance. The speed fluctuation is not obvious, so the speed has little effect on the visual load. The change of illuminance plays a leading role in the visual load, which shows that attention should be paid to the influence of the black-hole effect on visual load in the approach section. The lighting design in this section is particularly important, and the influence of environmental illumination outside the tunnel on the lighting inside the tunnel should be considered.

At the entrance and exit sections, a driver’s visual load is positively correlated with the velocity and negatively correlated with the illuminance, because, with the decrease of velocity and the increase of illuminance, the driver has better recognition and a better sight distance, so the visual load is lower, which is consistent with existing research. The change of velocity has a more obvious influence on the visual load, which indicates that the speed of the road should be strictly limited. It is suggested to strengthen the velocity control through engineering measures and management methods such as monitoring and deceleration marking.

When driving in the departure section, the visual load generally decreases first and then increases with the increase of velocity and illuminance. This is because, owing to the long time load accumulation in an extra-long tunnel, the load is quickly released after leaving the tunnel. Although the speed is increasing and affected by the white-hole effect, the visual load has declined. When the driver adapts, the visual load begins to increase with speed. Hence the coupling effect of speed management and lighting design should be comprehensively considered in this section.

The advantage of GA-SVR method is the better prediction effect of machine learning compared with multiple linear analysis, but the disadvantage is lacking the analysis of temporal and spatial dynamic evolution characteristics of optimization parameters. In fact, when GA-SVR analyzes tunnel segmentation to determine the optimal parameters of SVR, tunnel segmentation had to be divided according to the experience of researchers. Therefore, in the future study, it is necessary to further explore the segmentation method of long mountain tunnels from the perspective of data drive, and analyze the characteristics of temporal and spatial dynamic evolution of optimization parameters.

## 6. Conclusion

Based on velocity and illuminance coupling, a visual load model was constructed using the optimized support vector machine (GA-SVM). The influence of velocity and illuminance on the MTPA in the tunnel’s approach, entrance, exit, and departure section was analyzed. The following are the main conclusions of this study:

1. Different from the rule that the black-hole effect of a long tunnel is stronger than the white-hole effect, owing to the long-term accumulation of visual load in an extra-long tunnel, the driver’s psychological tension in the exit section is ranked as follows: entrance section ≈ exit section > departure section > approach section.
2. A GA-SVR model was used to analyze the changing relationship between MTPA, velocity, and illuminance in four entrance and exit sections of an extra-long tunnel. Compared with the traditional cross-search and network search methods, GA can optimize SVR parameters, and can improve accuracy from 89% to 96%.
3. In the approach section, the change of illuminance plays a leading role in the visual load, and a focus should be placed on the influence of environmental illumination outside the tunnel on the lighting in the tunnel. In the entrance and exit sections, the visual load is positively correlated with velocity and negatively correlated with illuminance. The change of velocity has a more obvious influence on the visual load. It is suggested that the control of driving speed should be strengthened through engineering measures and management methods such as monitoring and deceleration marking. In the departure section, the influence of the two variables on the driving visual load is synergistic, and the coupling effect of speed management and lighting design must be comprehensively considered.

Some scholars have studied the driver’s visual load in long tunnels and urban tunnels. However, expressway long tunnels, urban extra-long tunnels, and expressway extra-long tunnels have different speed limits, traffic composition, and lighting. No research has been conducted on the safety of visual loads at the entrance and exit of an expressway extra-long tunnel based on optimized SVR. The main limitation of the present study is that it only analyzes a tunnel with a straight line in the plane, without considering the influence of the curve and slope on the driver’s visual load. The prediction effect of GA-SVR is better than that of multiple linear analysis. If the extra-long mountain tunnels are further divided from the perspective of the data-driven, it can help to analyze the spatiotemporal dynamic evolution characteristics of parameters. In addition, this study does not consider the influence of weather conditions on visual loads, so the effect and mechanism of weather factors on visual loads require further analysis. This study only considers driver’s visual load when driving in the extra-long tunnel. However, on the mountainous highway, the driving risk of the truck is higher than that of the car. Therefore, in future research, we should pay attention to the influence of the extra-long tunnel on truck drivers’ visual load.

## Supporting information

S1 Data (PDF)

S2 Data (PDF)
