# Supplementary material for: Safety evaluation of visual load at entrance and exit of extra-long expressway tunnel based on optimized support vector regression
Source: PLoS One. 2022 Aug 4;17(8):e0272564. doi: 10.1371/journal.pone.0272564 (PMC9352028; doi:10.1371/journal.pone.0272564)
Supplement: S1 Data — (PDF) [file pone.0272564.s001.pdf]

| | Entrance（%） | Exit（%） | | |
|---|---|---|---|---|
| 1 | 4.34 | 17.78 | -200 | 200m Before the Entrance/Exit |
| 2 | 3.70 | 19.94 | -195 | |
| 3 | 4.24 | 20.83 | -190 | |
| 4 | 5.04 | 19.78 | -185 | |
| 5 | 7.85 | 20.58 | -180 | |
| 6 | 8.23 | 19.16 | -175 | |
| 7 | 9.04 | 17.31 | -170 | |
| 8 | 8.04 | 15.48 | -165 | |
| 9 | 7.66 | 16.45 | -160 | |
| 10 | 7.85 | 16.64 | -155 | |
| 11 | 7.48 | 19.31 | -150 | |
| 12 | 10.47 | 19.38 | -145 | |
| 13 | 9.12 | 18.94 | -140 | |
| 14 | 11.29 | 19.16 | -135 | |
| 15 | 13.12 | 18.78 | -130 | |
| 16 | 13.48 | 20.21 | -125 | |
| 17 | 15.32 | 18.56 | -120 | |
| 18 | 12.08 | 21.03 | -115 | |
| 19 | 9.75 | 18.41 | -110 | |
| 20 | 14.83 | 18.62 | -105 | |
| 21 | 16.91 | 20.23 | -100 | |
| 22 | 13.29 | 22.04 | -95 | |
| 23 | 12.29 | 27.85 | -90 | |
| 24 | 10.12 | 18.04 | -85 | |
| 25 | 10.84 | 22.85 | -80 | |
| 26 | 14.95 | 25.04 | -75 | |
| 27 | 17.47 | 23.04 | -70 | |
| 28 | 19.29 | 27.48 | -65 | |
| 29 | 17.12 | 27.66 | -60 | |
| 30 | 16.66 | 21.60 | -55 | |
| 31 | 18.66 | 25.70 | -50 | |
| 32 | 18.48 | 27.85 | -45 | |
| 33 | 17.23 | 27.48 | -40 | |
| 34 | 20.04 | 30.66 | -35 | |
| 35 | 15.04 | 31.66 | -30 | |
| 36 | 20.21 | 32.66 | -25 | |
| 37 | 22.62 | 33.55 | -20 | |
| 38 | 23.85 | 31.47 | -15 | |
| 39 | 25.58 | 33.75 | -10 | |
| 40 | 26.00 | 34.91 | -5 | |
| 41 | 23.78 | 36.90 | 0 | Entrance/Exit |
| 42 | 26.62 | 40.91 | 5 | |
| 43 | 30.04 | 43.08 | 10 | |
| 44 | 32.04 | 36.75 | 15 | |
| 45 | 33.85 | 40.33 | 20 | |
| 46 | 38.85 | 39.79 | 25 | |
| 47 | 40.48 | 35.91 | 30 | |

| | | | | |
|---|---|---|---|---|
| 48 | 37.49 | 30.85 | 35 | |
| 49 | 34.64 | 36.68 | 40 | |
| 50 | 32.47 | 30.61 | 45 | |
| 51 | 28.91 | 33.37 | 50 | |
| 52 | 27.08 | 31.38 | 55 | |
| 53 | 30.75 | 26.70 | 60 | |
| 54 | 21.09 | 20.81 | 65 | |
| 55 | 25.27 | 28.23 | 70 | |
| 56 | 22.29 | 28.04 | 75 | |
| 57 | 17.47 | 25.23 | 80 | |
| 58 | 20.12 | 21.04 | 85 | |
| 59 | 17.29 | 22.04 | 90 | |
| 60 | 21.66 | 18.23 | 95 | |
| 61 | 20.48 | 18.23 | 100 | |
| 62 | 17.47 | 18.04 | 105 | |
| 63 | 15.29 | 20.18 | 110 | |
| 64 | 20.29 | 19.23 | 115 | |
| 65 | 27.29 | 19.91 | 120 | |
| 66 | 24.47 | 13.50 | 125 | |
| 67 | 20.60 | 23.00 | 130 | |
| 68 | 21.47 | 18.04 | 135 | |
| 69 | 23.29 | 17.85 | 140 | |
| 70 | 22.80 | 17.66 | 145 | |
| 71 | 16.75 | 19.66 | 150 | |
| 72 | 17.91 | 20.29 | 155 | |
| 73 | 20.91 | 17.47 | 160 | |
| 74 | 16.91 | 17.45 | 165 | |
| 75 | 20.53 | 15.29 | 170 | |
| 76 | 16.91 | 16.29 | 175 | |
| 77 | 16.75 | 14.29 | 180 | |
| 78 | 12.08 | 13.29 | 185 | |
| 79 | 17.29 | 17.47 | 190 | |
| 80 | 11.77 | 12.57 | 195 | |
| 81 | 14.21 | 15.66 | 200 | 200 m After the Entrance/Exit |
