# Supplementary material for: Safety evaluation of visual load at entrance and exit of extra-long expressway tunnel based on optimized support vector regression
Source: PLoS One. 2022 Aug 4;17(8):e0272564. doi: 10.1371/journal.pone.0272564 (PMC9352028; doi:10.1371/journal.pone.0272564)
Supplement: S2 Data — (PDF) [file pone.0272564.s002.pdf]

	MTPA（mm2/s）	Velocity（m/s）	lluminance（cd/m2）	Location（m）			
1	4.26	101.3	4875.6	-200	200m Before the Entrance		
2	3.72	103.5	4875.6	-195			
3	2.59	98.6	4875.6	-190			
4	3.4	100.1	4875.6	-185			
5	4.32	99.2	4875.6	-180			
6	3.89	95.2	4702.2	-175			
7	3.25	103.4	4702.2	-170			
8	4.68	101	4702.2	-165			
9	5.37	98.8	4702.2	-160			
10	5.64	101.2	4816.3	-155			
11	5.15	96.4	4816.3	-150			
12	3.77	96.5	4816.3	-145			
13	3.21	100.2	4816.3	-140			
14	4.23	99.7	4725.6	-135			
15	5.6	97.3	4725.6	-130			
16	3.22	97.7	4725.6	-125			
17	3.13	97.2	4725.6	-120			
18	4.84	96.6	4730.8	-115			
19	4.17	95.3	4730.8	-110			
20	5.38	92.2	4730.8	-105			
21	5.57	93.1	4730.8	-100			
22	6.43	91	4681.2	-95			
23	3.12	92.3	4681.2	-90			
24	4.65	90.5	4681.2	-85			
25	4.38	89.9	4681.2	-80			
26	3.03	89.3	4595.5	-75			
27	7.43	86.2	4595.5	-70			
28	7.11	86.1	4595.5	-65			
29	8.93	87.1	4595.5	-60			
30	4.24	84.9	4413.7	-55			
31	5.31	83.4	4413.7	-50			
32	6.18	80.3	4413.7	-45			
33	8.64	75.5	4413.7	-40			
34	5.21	74.8	3874	-35			
35	6.68	75.8	3874	-30			
36	7.36	74.2	3874	-25			
37	7.48	74.9	3874	-20			
38	5.39	74.2	2126.9	-15			
39	6.14	75.5	2126.9	-10			
40	6.72	76.6	2126.9	-5			
41	8.24	77.2	216.2	0	Entrance		
42	7.67	76.3	216.2	5			
43	8.15	75.7	216.2	10			
44	9.64	77.8	216.2	15			
45	11.93	76.2	163.8	20			
46	12.56	77.1	163.8	25			
47	12.36	72.4	163.8	30			

48	9.91	73.1	163.8	35			
49	9.05	73	163.8	40			
50	10.28	75.6	110.5	45			
51	9.19	72.8	110.5	50			
52	9.59	74.9	110.5	55			
53	10.46	76.4	110.5	60			
54	10.09	73.3	82.4	65			
55	8.21	71.8	82.4	70			
56	9.26	72.1	82.4	75			
57	11.52	70.3	82.4	80			
58	12.18	73.6	82.4	85			
59	14.35	73.2	82.4	90			
60	14.87	75.4	61.1	95			
61	13.86	78.7	61.1	100			
62	8.45	72.4	61.1	105			
63	8.94	69.5	61.1	110			
64	13.45	70.3	25.6	115			
65	15.81	75.1	25.6	120			
66	19.65	71.2	25.6	125			
67	18.77	70.3	25.6	130			
68	17.38	73.5	25.6	135			
69	12.5	74.6	27.7	140			
70	12.06	72.7	27.7	145			
71	13.24	67.2	27.7	150			
72	18.18	76	27.7	155			
73	23.64	73.6	26.1	160			
74	25.57	75.5	26.1	165			
75	27.02	78.2	26.1	170			
76	24.19	78.7	26.1	175			
77	26.46	77.3	8.4	180			
78	28.38	75.9	8.4	185			
79	27.16	75.4	8.4	190			
80	26.27	75.5	8.4	195			
81	25.34	76.1	8.4	200	200m After the Entrance		
82	18.16	73.3	3.3	4545	200m Before the Exit		
83	19.73	73.5	3.3	4550			
84	20.49	71.6	3.3	4555			
85	20.07	77.2	3.3	4560			
86	16.46	70.9	3.5	4565			
87	15.25	79.2	3.5	4570			
88	16.75	79.9	3.5	4575			
89	17.3	75.7	3.5	4580			
90	18.22	79.5	3.3	4585			
91	19.49	72.2	3.3	4590			
92	16.51	74.3	3.3	4595			
93	16.23	69.1	3.3	4600			
94	20.54	74	3.6	4605			

95	20.82	72.2	3.6	4610			
96	17.92	68.3	3.6	4615			
97	17.28	74.1	3.6	4620			
98	18.95	71.2	3.4	4625			
99	18.41	67.8	3.4	4630			
##	17.85	78.7	3.4	4635			
##	18.69	71.3	3.4	4640			
##	17.77	66.4	3.2	4645			
##	15.91	76.6	3.2	4650			
##	14.07	70.3	3.2	4655			
##	17.24	71.2	3.2	4660			
##	20.39	73.4	3.2	4665			
##	20.35	69.8	3.2	4670			
##	23.48	70.5	7.1	4675			
##	26.12	75.6	7.1	4680			
##	25.05	69.7	7.1	4685			
##	18.74	73.5	7.1	4690			
##	16.42	68.9	9.4	4695			
##	15.73	76.2	9.4	4700			
##	17.88	67.1	9.4	4705			
##	17.14	71.3	9.4	4710			
##	18.65	65.2	15.8	4715			
##	20.73	76.1	15.8	4720			
##	23.89	69.2	15.8	4725			
##	22.02	71.3	15.8	4730			
##	27.91	79.7	15.8	4735			
##	30.54	77.4	154.2	4740			
##	31.26	75.5	154.2	4745	Exit		
##	31.24	74.1	154.2	4750			
##	32.33	70.6	3219.6	4755			
##	30.86	75.4	3219.6	4760			
##	28	67.7	3219.6	4765			
##	25.28	74.6	3219.6	4770			
##	25.56	70.9	3885.1	4775			
##	22.47	71.7	3885.1	4780			
##	22.83	73.8	3885.1	4785			
##	25.53	69.5	3885.1	4790			
##	29.67	72.4	4605.6	4795			
##	32.23	76.3	4605.6	4800			
##	31.15	72	4605.6	4805			
##	42.04	72.9	4605.6	4810			
##	43.23	73.4	4322.7	4815			
##	40.78	76.5	4322.7	4820			
##	37.26	77.2	4322.7	4825			
##	30.52	81.3	4322.7	4830			
##	29.89	88.7	4447.3	4835			
##	30.05	82.4	4447.3	4840			
##	32.25	85.1	4447.3	4845			

##	33.27	87.7	4447.3	4850			
##	27.57	93.3	4587	4855			
##	22.69	90.2	4587	4860			
##	20.31	85.9	4587	4865			
##	24.24	92.7	4587	4870			
##	25.3	92.4	4692.6	4875			
##	24.33	95.1	4692.6	4880			
##	20.72	95.5	4692.6	4885			
##	25.39	103.9	4692.6	4890			
##	17.59	105.8	4811.9	4895			
##	20.76	107.4	4811.9	4900			
##	21.27	109.2	4811.9	4905			
##	23.36	115.3	4811.9	4910			
##	22.48	110.1	4675.8	4915			
##	17.74	111.8	4675.8	4920			
##	16.38	111.6	4675.8	4925			
##	15.76	113.4	4675.8	4930			
##	12.58	108.5	4675.8	4935			
##	10.16	107.3	4675.8	4940			
##	10.07	116.6	4675.8	4945	200m After the Exit		
